# Map-based cloning and functional analysis of *YGL8*, which controls leaf colour in rice (*Oryza sativa*)

**DOI:** 10.1186/s12870-016-0821-5

**Published:** 2016-06-13

**Authors:** Xiaoyan Zhu, Shuang Guo, Zhongwei Wang, Qing Du, Yadi Xing, Tianquan Zhang, Wenqiang Shen, Xianchun Sang, Yinghua Ling, Guanghua He

**Affiliations:** Key Laboratory of Application and Safety Control of Genetically Modified Crops, Rice Research Institute of Southwest University, Chongqing, 400716 China; Institute of Rice, Chongqing Academy of Agricultural Sciences, Chongqing, 401329 China; Forestry Bureau of Chuanshan District, Suining, Sichuan 629000 China

**Keywords:** Rice (*Oryza sativa*), *Yellow-green leaf 8* (*ygl8*), Map-based cloning, Functional analysis

## Abstract

**Background:**

As the indispensable part of plant, leaf blade mainly functions as the production workshops where organic substance is produced by photosynthesis. Leaf colour mutation is a genetic phenomenon that has a high frequency and is easily identified. The mutations always exhibit negative impact on the development of plants in any of the different stages of growth. Up to now, numerous genes involved in leaf colour mutations have been cloned.

**Results:**

In this study, a yellow-green leaf mutant, *yellow-green leaf 8* (*ygl8*), with stable genetic phenotype, has been screened out in the progeny of an excellent *indica* restorer line Jinhui 10 with seeds treated by EMS. The levels of Chl *a*, Chl *b* and total chlorophyll were significantly lower in *ygl8* than those in the WT throughout the whole growth period, while no clear change was noted in the Chl *a*/*b* ratio. Transmission electron microscopy demonstrated that the lamellae were clearly intumescent and intricately stacked in *ygl8*. Furthermore, compared with those of the WT, the stomatal conductance, intercellular CO_2_ concentration, photosynthetic rate and transpiration rate of *ylg8* were all significantly lower. Map-based cloning results showed that *Loc_Os01g73450*, encoding a chloroplast-targeted UMP kinase, corresponded to Ygl8 and played an important role in regulating leaf colour in rice (*Oryza sativa*). Complementation of *ygl8* with the WT DNA sequence of *Loc_Os01g73450* led to restoration of the normal phenotype, and transgenic RNA interference plants showed a yellow-green colour. Analysis of the spatial and temporal expression of Ygl8 indicated that it was highly expressed in leaf blades and weakly expressed in other tissues. qRT-PCR also showed that the expression levels of the major Photosystem I core subunits plastome-encoded PsaA, PsaB and PsbC were significantly reduced in *ygl8*. The expression levels of nuclear-encoded gene involved in Chl biosynthesis HEMC, HEME, and PORA were also decreased when compared with the wild-type.

**Conclusions:**

Independent of Chl biosynthesis and photosystem, YGL8 may affect the structure and function of chloroplasts grana lamellae by regulating plastid genome encoded thylakoid membrane constitutive gene expression and indirectly influences Chl biosynthesis.

**Electronic supplementary material:**

The online version of this article (doi:10.1186/s12870-016-0821-5) contains supplementary material, which is available to authorized users.

## Background

Leaf colour mutation occurs commonly in the natural world. There are abundant phenotypes and the mutations can exhibit their effects in any of the different stages of growth. With regard to the classification of leaf colour mutants, numerous different categories have been established. Gustafsson classified leaf colour mutants into albino, chlorina, stripe, virescent and zebra [1]. Later, Awan et al. subdivided them into eight classes: albino, xantha, viridis, alboviridis, viridoalbino, xanthaviridis, viridoxantha and striata [2]. With regard to the physiological mechanisms involved, leaf colour mutations were divided by Falbel and Staehelin into total chlorophyll (Chl) increase type, total Chl deficiency type, Chl *a* deficiency type and Chl *b* deficiency type [3].

To date, the locations of more than 100 leaf colour mutations have been determined in rice and have been shown to be distributed across all 12 chromosomes. Over 40 genes involved in leaf colour mutations have been cloned, more than 10 of which were found to be chlorina genes. Examples of these include *OsHAP3A*, *OsHAP3B* and *OsHAP3C*, which are three yellowing genes that were shown to control chloroplast biosynthesis [4]. *OsCAO1* and *OsCAO2* were both found to encode Chl *a* oxygenase, which is the key enzyme for the catalytic synthesis of Chl *b* [5]. *Ygl1* was shown to encode Chl synthase, which catalyses conversion of chlorophyllide *a* into Chl *a*, the final step in the biosynthesis of Chl *a* [6]. *Vyl* encodes a protein homologous to the *Arabidopsis* caseinolytic protease P6 subunit and is targeted to the chloroplast [7]. *V2* was found to encode a new type of guanylate kinase (GK, ATP: GMP phosphotransferase), which is the key enzyme in guanine metabolic pathways and participates in regulating the transmission of chloroplast development signals to the nucleus [8]. *Ygl2* encodes a heme oxygenase, which is the initial and rate-limiting enzyme that catalyses heme degradation [9]. *V3* encodes the large subunit of ribonucleotide reductase, which controls the rate of synthesis of DNA nucleotides and affects DNA synthesis and repair [10]. There are also *CHL1* and *CHL9*, which respectively encode the ChlD and ChlI subunits of Mg-chelatase, a key enzyme for Chl synthesis and chloroplast development [11]. Newly identified *Ygl6* gene encoding a putative 3-β-hydroxysteroid dehydrogenase/isomerase family protein might be involved in the synthesis of brassinosteroids, and it shows sequence similarity to human and mouse proteins involved in the synthesis of androgens, progestins, and estrogens [12]. Recently, another gene, *Ygl7*, an allele of the *OsChlD* gene, was identified. The *ygl7* mutant uses light energy efficiently when compared with the wild-type, which demonstrated that the *YGL7* protein might promote photosynthesis [13]. Besides *V2*, *V3*, and *Ygl6*, other cloned leaf colour mutation genes were almost directly involved in Chl metabolism or chloroplast development.

Pyrimidine nucleotides represent one of the most fundamental cellular components with essential functions in multiple cellular processes. They are not only building blocks for nucleic acid synthesis in dividing and elongating tissues, but also participate in lipid and carbon metabolism such as UDP-glucose as precursor in sucrose or cell wall biosynthesis. Pyrimidine nucleotides are synthesized both from amino acids and other small molecules via *de novo* pathways, and from preformed nucleobases and nucleosides by salvage pathways. UMP kinase which catalyses the phosphorylation of UMP to UDP, with ATP as the preferred donor [24] plays a crucial role in pyrimidine nucleotides de novo synthesis and functions in supplying precursors for nucleic acid synthesis by catalyzing the conversion of UMP into their diphosphate form. The functions of UMP kinase in cell proliferation and physiology have been widely studied. It has been reported that in both bacteria and yeast, the mutation of UMP kinase represses cell proliferation and physiology [14, 15]. In addition, Giblett et al. [16] has reported that the phenotype of down-regulated UMP kinase was similar to the immune defect resulting from adenosine deaminase deficiency. However, in plants, only a few studies on UMP kinase have been carried out [17, 18]. Zhou et al. has demonstrated the Arabidopsis thaliana UMP/CMP kinase activity by complementation of a *Saccharomyces cerevisiae ura6* mutant [17]. The mutation of an Arabidopsis prokaryotic UMP kinase has defects in *psaA/B* transcript accumulation and leads to growth and development retardation of the mutant plants [18].

To date, numerous proteins involved in Chl biosynthesis and assembly and regulation of the photosystems have been identified, greatly broadening our understanding of their function in crop yield. Our knowledge about the function of UMP kinase in rice and its function in regulating the photosystems and Chl biosynthesis is still limited. Here, we report the identification of *ygl8* (*yellow-green leaf 8*), a new leaf colour mutant in *O. sativa*. The *ygl8* mutant exhibits abnormal grana lamellae and a yellow-green phenotype. YGL8 protein is a nuclear encoded chloroplast targeted protein which shares high sequence similarity with cyanobacterial UMP kinase. Possible roles of YGL8 in regulating photosystem and Chl biosynthesis are discussed.

## Results

### The *Ygl8* gene mutation led to less Chl accumulation and destroyed the structure of grana lamellae

**Fig. 1:**
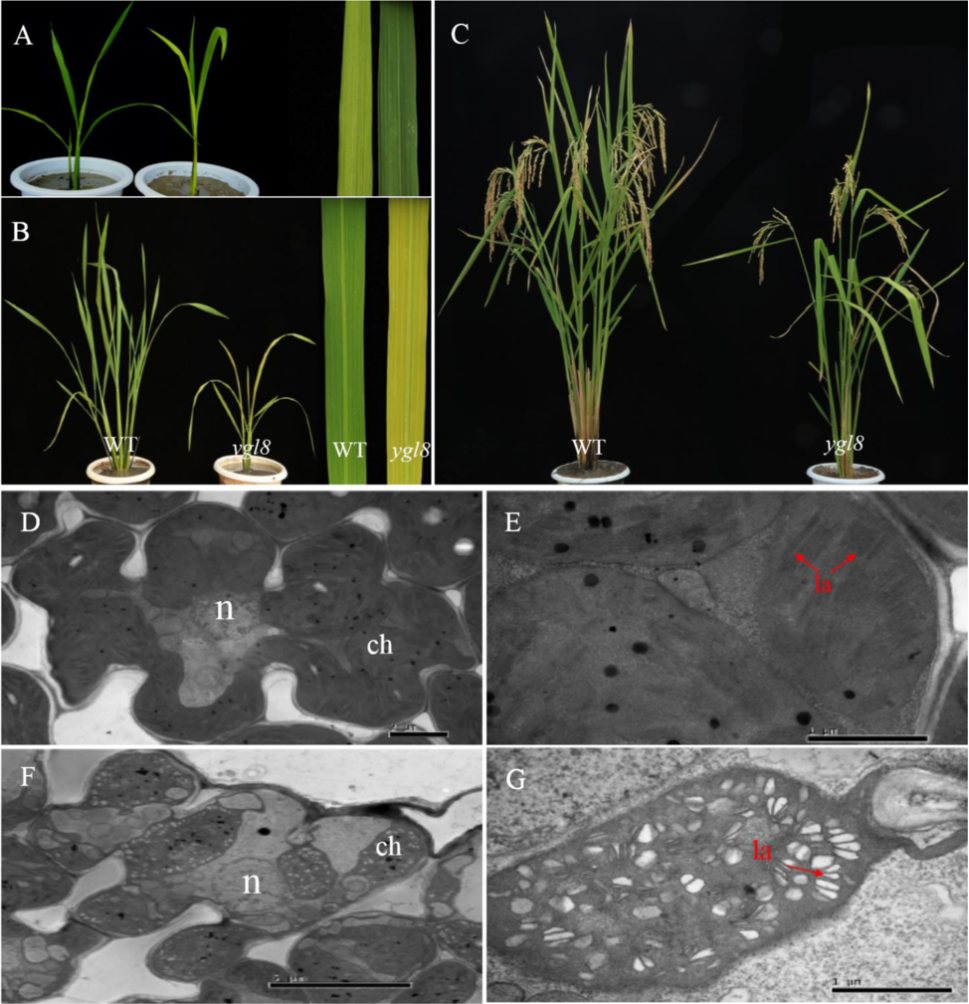
The phenotype, cytology and agronomic traits of the wild-type and *ygl8* mutant. **a**: The phenotype at the seedling stage; **b**: the phenotype at the tillering stage; **c**: the phenotype at the mature stage; **d**–**e**: cytological analysis of the WT; **f**–**g**: cytological analysis of *ygl8*; ch: chloroplast; la: grana lamella; n: nucleus

A yellow-green leaf mutant, *ygl8*, derived from a process of ethyl methane sulfonate (EMS) mutagenesis and exhibiting a yellow-green leaf phenotype throughout the entire growth period, was identified (Fig. 1a–c). Its level of Chl was significantly decreased throughout the whole growth period, especially at the tillering stage when it was only 37.30 % of the normal (wild-type; WT) level. This mutant also had Chl reductions of 59.24 % at the seedling stage and 57.47 % at the heading stage. More specifically, the levels of both Chl *a* and Chl *b* were significantly lower in *ygl8* than those in the WT throughout the whole growth period. However, the ratio of Chl *a*/*b* changed little between the *ygl8* mutant and the wild-type, with a slight increase in *ygl8* at the seedling and tillering stages. The decrease in Chl level corresponded perfectly to the level of yellowness of the leaves (Table 1).

**Table 1:** Levels of various photosynthetic pigments in leaves of the mutant and the wild-type at the seedling, tillering and heading stages

		Chl *a* (mg g^−1^ FW)	Chl *b* (mg g^−1^ FW)	Total Chl (mg g^−1^ FW)	Chl *a*/*b* ratio
Seedling stage	WT	3.03 ± 0.13	0.60 ± 0.03	3.63 ± 0.15	5.05 ± 0.23
	*ygl8*	1.25 ± 0.14^**^	0.23 ± 0.03^**^	1.48 ± 0.17^**^	5.44 ± 0.24
Tilling stage	WT	3.40 ± 0.10	0.69 ± 0.08	4.09 ± 0.13	4.93 ± 0.18
	*ygl8*	1.29 ± 0.03^**^	0.23 ± 0.01^**^	1.53 ± 0.08^**^	5.60 ± 0.29
Heading stage	WT	3.55 ± 0.26	0.83 ± 0.06	4.38 ± 0.32	4.29 ± 0.06
	*ygl8*	1.48 ± 0.14^**^	0.38 ± 0.12^**^	1.86 ± 0.23^**^	4.10 ± 0.79

Fw indicates fresh weight; ** means significantly different compared with the corresponding WT at *p* < 0.01 by the Student’s *t* test

To investigate whether the *ygl8* mutation affects chloroplast development, we compared the ultrastructure of chloroplasts in *ygl8* and the WT at the seedling stage using *TEM*. There were normal cells and chloroplasts in the WT (Fig. 1d–e). We also found that the cells in *ygl8* maintained their structural integrity (Fig. 1f); however, the grana lamellae of chloroplasts were clearly intumescent and intricately stacked in *ygl8* (Fig. 1g).

**Fig. 2:**
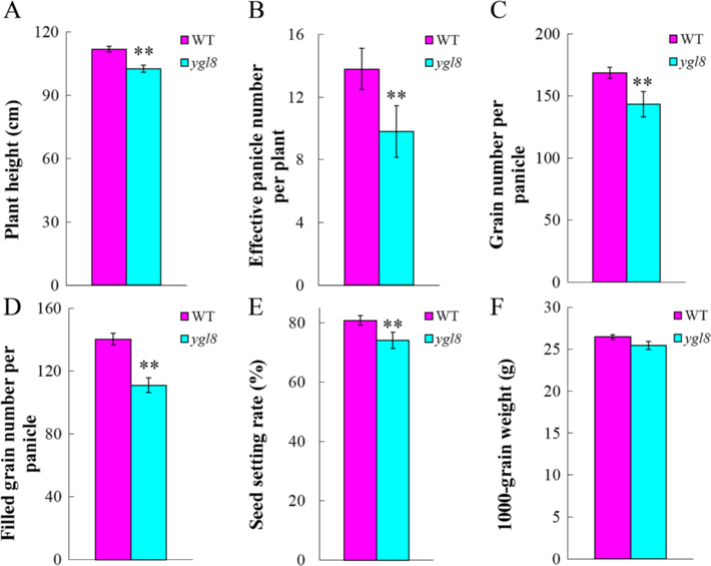
The agronomic traits of the wild-type and *ygl8* mutant. **a**–**f**: the main agronomic traits of WT and *ygl8.* Error bars represent standard deviation (SD) of three biological repeats; ** indicates significantly different compared with the corresponding WT at *p* < 0.01 by the Student’s *t* test

Most of the main agronomic traits were significantly worse in *ygl8* than those in the WT, especially plant height, effective panicle number per plant, grain number per panicle, filled grain number per panicle, and seed setting rate; however, 1000-grain weight showed no clear difference (Fig. 2a–f; Additional file 1).

### Photosynthetic capacity was affected by the mutation of *YGL8*

**Fig. 3:**
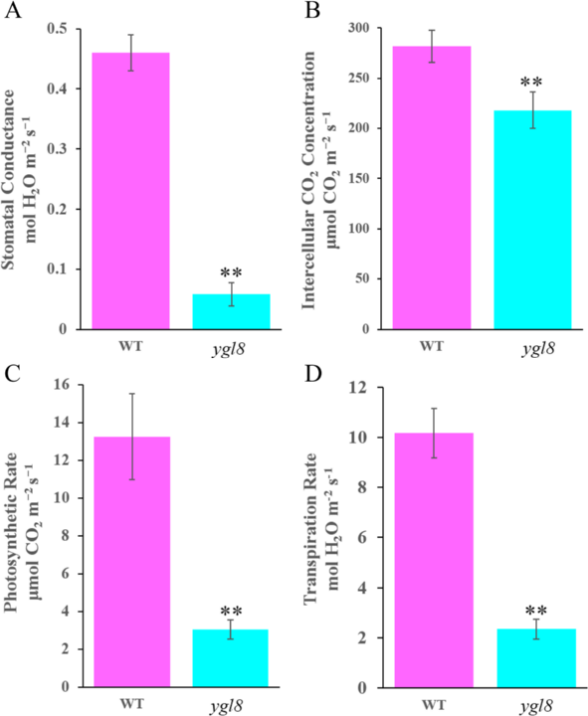
Comparison of stomatal conductance, intercellular CO_2_ concentration, photosynthetic rate and transpiration rate between WT and *ygl8* at the heading stage. Error bars represent standard deviation (SD) of three biological repeats; ** indicates significantly different compared with the corresponding WT at *p* < 0.01 by the Student’s *t* test

Photosynthetic parameters were analysed at the heading stage using an LI-6400 portable photosynthesis meter (LiCor, Lincoln, NE, USA). In *ygl8*, the stomatal conductance was just 0.058 mol H_2_O m^−2^ s^−1^, which was only 12.52 % of the value in the WT (Fig. 3a; Additional file 2), and the intercellular CO_2_ concentration was 218.22 μmol CO_2_ m^−2^ s^−1^ in *ygl8* but 281.75 μmol CO_2_ m^−2^ s^−1^ in the WT (Fig. 3b; Additional file 2). The photosynthetic rate was significantly decreased in *ygl8* compared with that in the WT (Fig. 3c; Additional file 2). Furthermore, the transpiration rate was 2.32 mol H_2_O m^−2^ s^−1^, significantly lower than that of the WT (Fig. 3d; Additional file 2). All of these findings indicated that the photosynthetic capacity was deficient in *ygl8*.

### The *Ygl8* gene encodes a UMP kinase on chromosome 1

**Fig. 4:**
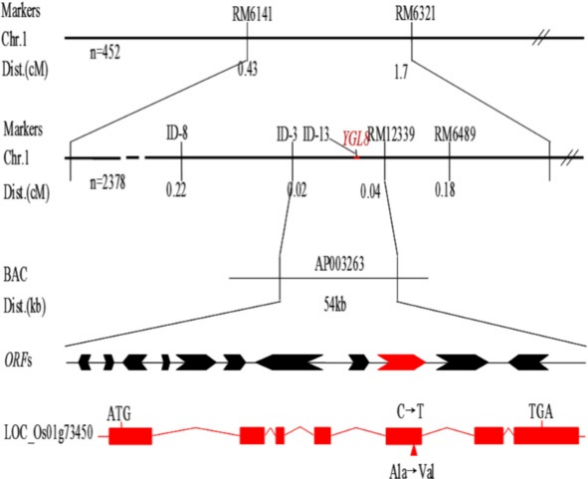
Map-based cloning and characterisation of *YGL8. YGL8* was located on chromosome 1 between In-Del marker ID-3 and SSR marker RM12339 and co-segregated with In-Del marker ID-13

**Fig. 5:**
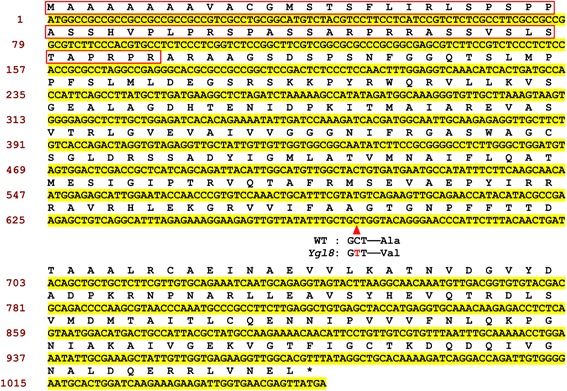
The coding sequences and amino acids of the YGL8 protein. The red box indicates the predicted chloroplast signal peptide and the base marked with a red triangle shows the mutation site of *ygl8*

Genetic analysis showed that the yellow leaf phenotype in *ygl8* is controlled by a single recessive nuclear locus with a segregation ratio of 2.987 (*χ*^2^ = 0.028 < *χ*^2^_0.05_ = 3.84). To map the *Ygl8* locus, 400 pairs of SSR markers evenly distributed in the rice genome were selected to screen for polymorphism among the parents and the gene pools. RM6141 and RM6321 revealed polymorphism among the parents and the gene pools, respectively. By using 452 F_2_ recessive plants, the *YGL8* gene was first mapped to the long arm of chromosome 1, between RM6141 and RM6321. To narrow down its location, more than 9000 F_2_ plants, of which 2360 were recessive, were used for fine mapping. More than 40 In-Del markers and SSR markers were designed, five of which were polymorphic. Finally, we mapped the *Ygl8* gene to a 54-kb genomic region between In-Del marker ID-3 and SSR marker RM12339, and Ygl8 co-segregated with In-Del marker ID-13 (Fig. 4). Within the target region, 11 ORFs were identified using the Gramene website (http://www.gramene.org/). Among the 11 ORFs sequenced, only *Loc_Os01g73450* carried a mutation, a single-nucleotide transition (C → T), on the fifth exon at the position 671 bp from the ATG start codon, which resulted in an amino acid change from Ala-224 to Val (Fig. 4) when compared with the wild-type. A search of the rice genome database reveals that *Loc_Os01g73450* consists of seven exons and six introns, with a total length of 4177 bp. The 1056 bp CDS encodes a UMP kinase with 351 amino acids (Fig. 5). Therefore, we tentatively assigned *Loc_Os01g73450* as the *Ygl8* gene.

**Fig. 6:**
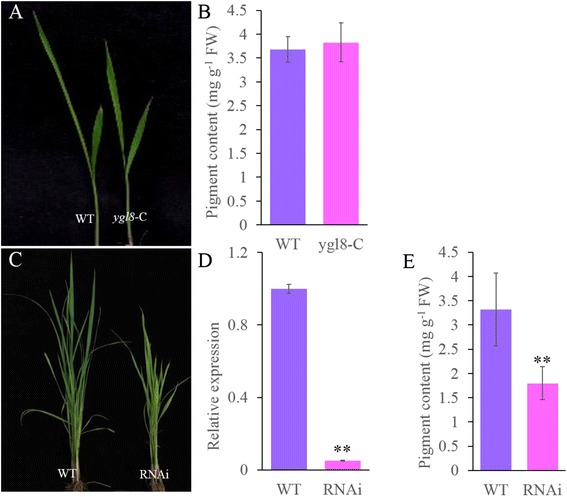
Identification of *YGL8*. **a**: the phenotype of the WT and a complementation (*ygl8*-C) plant; **b**: the total level of Chl in WT and *ygl8*-C plants; **c**: the phenotype of the WT (ZhongHua11) and a RNAi plant; **d**: the expression levels of *YGL8* in WT (ZhongHua11) and RNAi plants; **e**: the total level of Chl in WT (ZhongHua11) and RNAi plants; error bars represent standard deviation (SD) of three biological repeats; ** indicates significantly different compared with the corresponding WT at *p* < 0.01 by the Student’s *t* test

For the further validation of *Ygl8*, genetic complementation was carried out by introducing a 6478 bp genomic fragment of WT into *ygl8* plants. As expected, the yellow-green leaf phenotype was not observed in any *ygl8*-C transgenic line (Fig. 6a). Furthermore, the level of Chl was restored to the level in the WT (Fig. 6b). Confirming that disruption of the *YGL8* gene was responsible for the *ygl8* mutant phenotype, RNAi transgenic plants showed a yellow-green leaf phenotype in which the total Chl level was clearly decreased (Fig. 6c and e). qRT-PCR analysis revealed that the expression of *YGL8* was markedly reduced in these transgenic lines compared with that in ZhongHua11 plants (Fig. 6d). Together, these results confirmed that *Loc_Os01g73450* indeed corresponded to the Ygl8 gene.

### YGL8 protein belongs to a highly conserved protein family

**Fig. 7:**
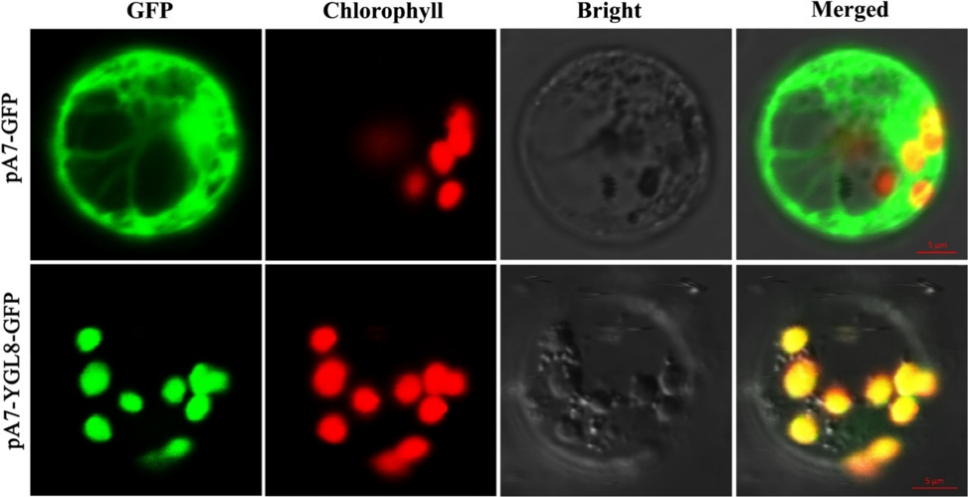
Subcellular location of the YGL8 protein. YGL8 protein was targeted to the chloroplast

**Fig. 8:**
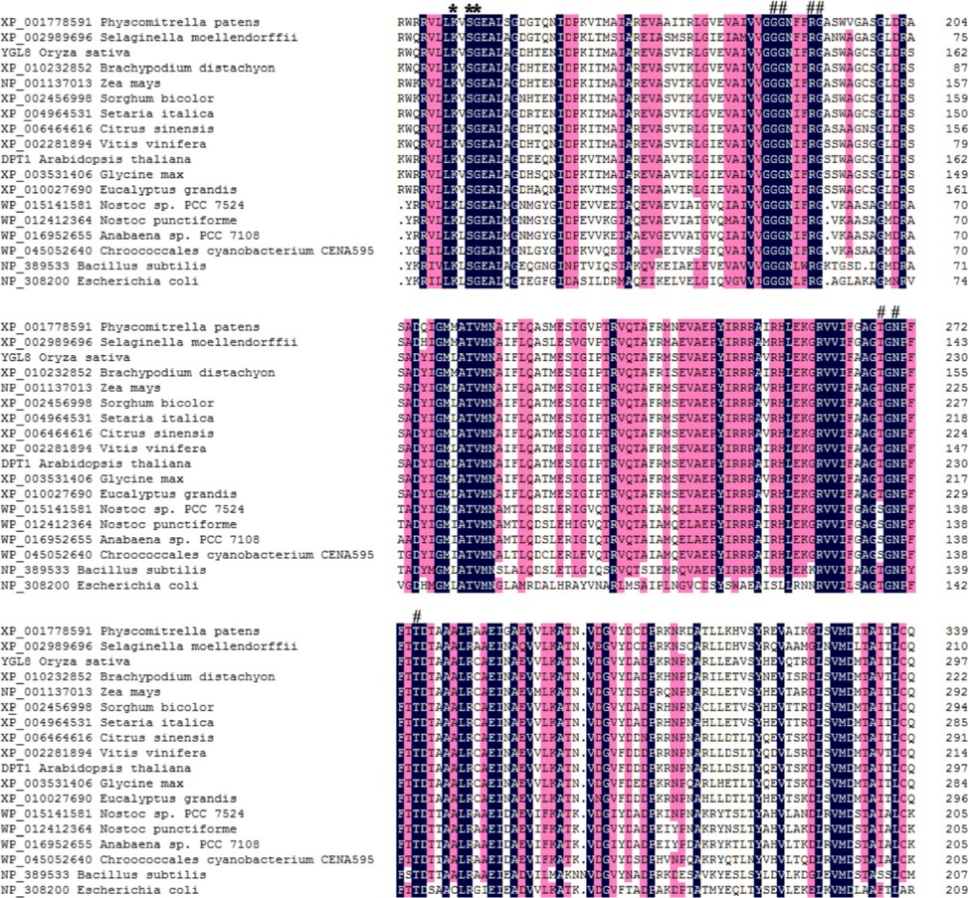
Multiple sequence alignment of the UMP kinase protein. The alignment of UMP kinase homologous proteins was calculated using the ClustX program. Blue background indicates 100 % homology of the aligned sequence; pink background indicates positions conserved in >75 % of the aligned sequences. # indicates residues known to interact with UMP; * indicates residues known to interact with either UDP or UTP [18]

**Fig. 9:**
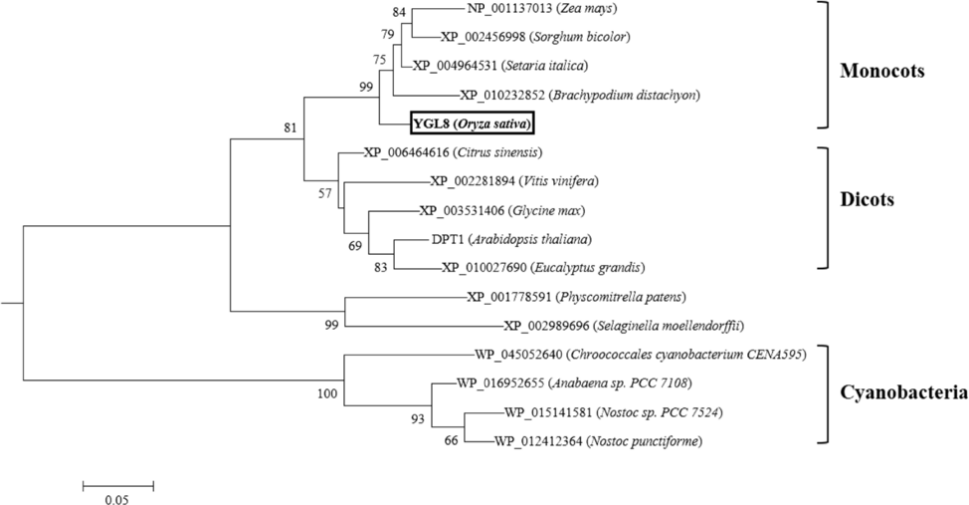
Phylogenetic tree of prokaryotic and eukaryotic UMP kinases. The tree for UMP kinase homologue amino acid sequences was calculated and constructed using the MEGA program [19]

The predicted YGL8 polypeptide consisted of 351 amino acids with a chloroplast transit sequence of 58 amino acids at the N-terminus. Thus, the mature protein had a predicted molecular weight of approximately 31.1 kDa. Subcellular location analysis in the rice protoplast indicated that the YGL8 protein functioned in chloroplasts (Fig. 7). Multiple sequence alignment and phylogenetic analysis indicated that YGL8 contained a conserved domain of a UMP kinase and shared significant similarity with other eukaryotic nuclear-encoded UMP kinases and their ancestral prokaryotic UMP kinases (Figs. 8 and 9). Most notably, in the conserved domain, YGL8 shared significant similarity with the conserved domain of cyanobacterial UMP kinases, including those of *Chroococcales cyanobacterium* CENA595, *Anabaena sp.* PCC 7108 and *Nostoc punctiforme* (Figs. 8 and 9).

### Expression patterns of *YGL8*

**Fig. 10:**
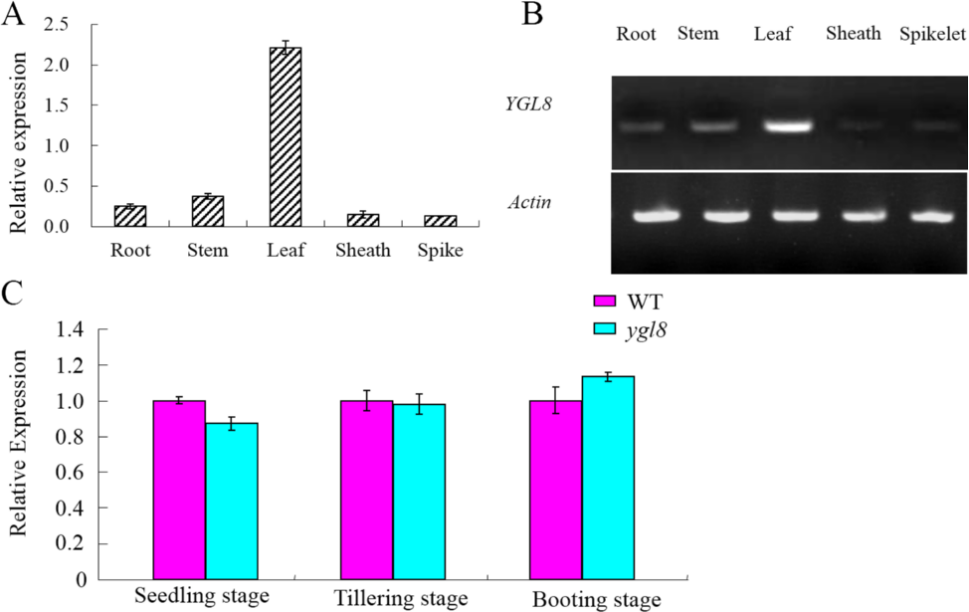
Expression of *YGL8* at different stages and in different tissues. **a**–**b**: Expression of *YGL8* in different tissues. **c**: Expression of *YGL8* in the first leaves from the top at different growth stages; **e**rror bars represent standard deviation (SD) of three biological repeats

qRT-PCR was applied to examine the expression pattern of *YGL8* in WT and *ygl8* mutant plants. At the booting stage, *YGL8* was strongly expressed in leaf blades, while it was weakly expressed in the other tissues examined (Fig. 10a), the same results were obtained by semi-qPCR (Fig. 10b). Furthermore, there was no clear difference in *YGL8* expression in leaf blades between the WT and the *ygl8* mutant at the seedling, tillering and booting stages (Fig. 10c), which may indicate that the single amino acid mutation from Ala to Val in the YGL8 protein caused by the single base mutation from C to T in the *YGL8* gene has no effect on the mRNA level.

### *YGL8* affected the expression of genes related to chloroplast development and the photosynthetic system

**Fig. 11:**
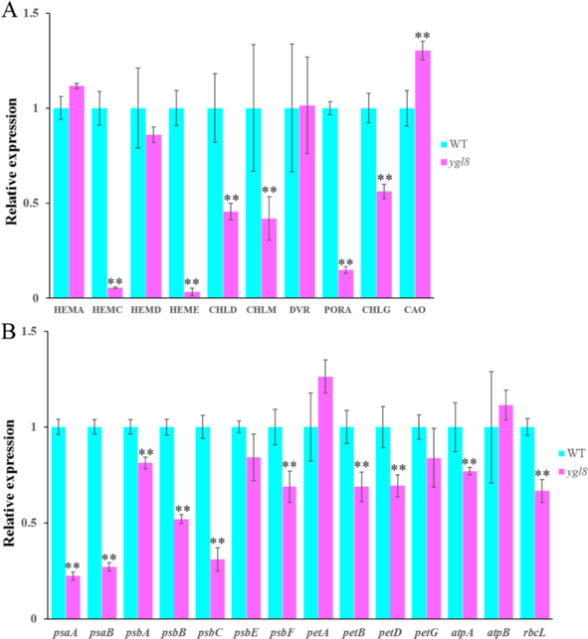
The expression of different genes. **a**: The expression of Chl biosynthesis-related genes; **b**: the expression of chloroplast genome encoded genes; error bars represent standard deviation (SD) of three biological repeats; ** indicates significantly different compared with the corresponding WT at *p* < 0.01 by the Student’s *t* test

We addressed the issue of whether the *ygl8* mutation affected the transcription of other genes associated with Chl biosynthesis and photosynthesis. The results of qRT-PCR showed that the expression levels of genes involved in Chl biosynthesis, such as *HEMC*, *HEME* and *PORA*, were significantly reduced, at only 5.47, 3.27 and 14.86 % of the levels in the WT, respectively, and the expression levels of *CHLD*, *CHLM* and *CHLG* were approximately half those in the WT. However, when compared with the wild type, the expression level of *CAO* was slightly increased in the *ygl8* mutant (Fig. 11a). Interestingly, in the *ygl8* mutant, most of the expression levels of chloroplast genome encoded genes which we have examined were significant lower than those of the wild type to a different extent. Especially, three genes encoding photosystem reaction centre polypeptides, *psaA*, *psaB*, and *psbC* were reduced in *ygl8*, at only 22.37, 27.07 and 30.9 % of the expression levels in the WT, respectively. In addition, the expression levels of *psbA*, *psbB*, *psbF*, *petB*, *petD*, *atpA*, and *rbcL* in the *ygl8* mutant were lower than those in the wild type, but the decreasing extent of corresponding gene was not high (Fig. 11b).

## Discussion

Recently, substantial research has shown that leaf colour mutants are ideal for shedding light on the mechanisms of plant photosynthesis, the Chl biosynthesis pathway, the structure and function of chloroplasts, and crop agronomic traits [20]. In this study, using fine mapping, we identified a yellow-green leaf gene, *YGL8*, which is located near the telomere of the long arm of chromosome 1 (Fig. 4) and encodes a UMP kinase (Figs. 5 and 8). No other mutated genes located in that area of chromosome 1 was identified by map-based cloning and none of the other cloned genes encoded a UMP kinase. Therefore, *YGL8* was identified as a novel gene that controls the development of leaf colour in rice.

### YGL8 protein may have evolved from cyanobacterial UMP kinase and regulate the UMP/UMP/UTP level in chloroplasts

It is generally accepted that all chloroplasts are derived from a single cyanobacterial ancestor via the process of endosymbiosis [21]. During evolution, almost all cyanobacterial genes retained by eukaryotes relocated to the cell nucleus [22]. Therefore, the vast majority of chloroplast proteins are now imported as products of nuclear genes that encode chloroplast-targeted precursors synthesised on cytosolic ribosomes [23]. Since nuclear-encoded *YGL8* is targeted to chloroplasts and shows high similarity to a homologous cyanobacterial protein, the *YGL8* gene might also have moved from the chloroplast to the nuclear genome of rice over the course of evolution. This would be consistent with the results of Puthiyaveetil et al*.* [21], who showed that the gene for the chloroplast sensor kinase moved from chloroplasts to the nuclear genome in *Arabidopsis* during evolution. Research has shown that in plants, as the building blocks of DNA and RNA synthesis, all pyrimidines within the cell are derived from UMP, [17]. UMP kinase is a ubiquitous enzyme that catalyses the phosphorylation of UMP to UDP, with ATP as the preferred donor [24]. The roles of pyrimidine monophosphokinases in cell growth and proliferation have been widely studied. For example, in *Escherichia coli*, the product of the UMP kinase gene (*pyrH/smbA*) has been shown to influence cell proliferation. The *smbA* mutant showed decreased macromolecular synthesis, hypersensitive to SDS and a novel morphological phenotype under nonpermissive conditions [14]. In addition, in yeast, mutations in the UMP kinase gene have been shown to cause declines of 90 % in UTP and CTP, which affects both RNA and protein synthesis and ultimately results in cell death [15]. In the model plant *Arabidopsis thaliana*, it was reported that mutants of chloroplast-targeted UMP kinase (DPT1) failed to grow photo-autotrophically [18]. Here, we identified that the *YGL8* gene encodes a chloroplast UMP kinase. The amino acid mutation from Ala to Val near the UMP binding site in the mutant protein (Figs. 4 and 5) might affect the enzyme activity of YGL8 and have a negative effect on the chloroplastic UTP pool, although this remains to be examined. These results indicate that nuclear-encoded and chloroplast-targeted YGL8, of cyanobacterial ancestry, may function in regulating the chloroplast UTP pool and influence chloroplast-encoded gene expression.

### Mutation of YGL8 indirectly affects the expression of some nuclear-encoded chlorophyll biosynthesis-related genes

Chlorophyll plays a significant role in plant development and crop yield [27]. Chl metabolism has been extensively studied in various organisms by both biochemical [28] and genetic approaches [29]. Mutants with a deficiency in the ability to synthesize Chl, including CHLH, CHLD, CHLI and CHLG, exhibit growth defects and/or reduced yield to different extents [5, 6, 11, 30]. In this study, we observed that the mutation of *YGL8* affected the mRNA expression of some genes involved in Chl biogenesis: among the genes tested, the expression of *HEMC*, *HEME*, *CHLD*, *CHLM*, *PORA* and *CHLG* was reduced to different extents (Fig. 11a). This might be the main factor that led to the decreased Chl *a* level in the *ygl8* mutant (Table 1). One of the most important components of the light-harvesting pigment protein complexes of PS I and PS II, Chl *b*, is synthesised by *CAO*, which was reported to convert chlorophyllide *a* (Chlide *a*) into Chlide *b*, most likely by two-step oxygenation [31]. Although the *ygl8* mutant retained a slightly increased mRNA level of *CAO* comparable to that of the WT (Fig. 11a), its Chl *b* level was markedly lower (Table 1). The main reason for this reduced Chl *b* content might be the significant blockage in Chl *a* biosynthesis. It has been reported that the ratio of Chl *a*/*b* is closely related to the mRNA levels of *CAO* in *Arabidopsis* [32]. However, the ratio of Chl *a*/*b* (Table 1) remained unchanged with a slightly increased CAO expression level. This might also be a consequence of decreased both Chl *a* and Chl *b* levels in the *ygl8* mutant. These results suggest that YGL8 may function in influencing Chl *a* and Chl *b* biosynthesis.

### Mutation of YGL8 affects the expression of some chloroplast-encoded genes

In eukaryotes, the two largest polypeptide subunits of PS I, PsaA and PsaB, both encoded by the plastome, form a heterodimer and bind to the primary electron donor of PS I, P700 [25]. It has been reported that mutants lacking the PsaA/B dimer generally failed to assemble the entire PS I core complex, although some peripheral subunits could still accumulate in the thylakoid membranes [26]. In the *ygl8* mutant, the level of *psaA* and *psaB* transcripts was found to be severely impaired (Fig. 11b), which implied that *ygl8* might also fail to assemble a functional PS I core complex, which would affect its photosynthesis. And this is consistent with the findings of Hein et al. [18], which showed that the *dpt1-1* mutant of *A. thaliana* failed to grow photoautotrophically because of a reduced *psaA* and *psaB* mRNA level. Three genes of the chloroplast genome, *psbA*, *psbB*, *psbC* and *psbF*, encoding D1 protein, CP47 protein, CP43 protein, and cytochrome b559 of the photosystem II reaction centre, respectively [39], exhibited different degrees of lower gene expression in the *ygl8* mutant (Fig. 11b). Besides, other plastome-encoded genes, *petB*, *petD*, *atpA*, and *rbcL*, encoding cytochrome b6, subunit IV of cytochrome *b*/*f* complex, ATPase alpha subunit, and rubisco large subunit, respectively [39], showed lower expression level in the *ygl8* mutant, in spite that the decreasing degree is not high (Fig. 11b). Thus, we suggest that the mutation of chloroplast-targeted UMP kinase in the *ygl8* mutant may affect the chloroplast UTP pool, which leads to the changing mRNA level of chloroplast genome encoded genes. Chloroplast thylakoid membranes are the main structure of energy transformation in plants. The four major functional membrane protein complexes of thylakoid membranes: the photosystem I and II, the cytochrome *b*/*f* complex, and ATPase, act cooperatively to transfer light energy into available chemical energy [40]. Reduced expression level of thylakoid membrane genes in the *ygl8* mutant which we have examined may lead to the abnormal structure (intumescent and hollow, Fig. 1f–g) and function (decreased photosynthetic rate, Fig. 3c; Additional file 2) of chloroplast thylakoid membranes. These results reveals that YGL8 may affect thylakoid membrane structure and function by influencing chloroplast genome encoded thylakoid membrane constitutive genes.

It is not yet fully understood why the mutation of chloroplast-targeted *YGL8* affects nuclear-encoded Chl biosynthesis-related genes dramatically, and ultimately leads to a decline in the level of Chl in the *ygl8* mutant. According to the recent reports and reviews on plastid-to-nucleus retrograde signaling, it is clear that the expression of nuclear genes for plastid proteins is affected by the development and/or metabolic state of chloroplast [41, 42]. Thus, one possible explanation might be that abnormal thylakoid membrane development resulted from the mutation of chloroplast-targeted UMP kinase by influencing the mRNA level of chloroplast genome encoded genes may inhibit Chl biosynthesis-related gene expression by plastid-to-nucleus retrograde signaling.

## Conclusions

In this study, a rice leaf yellowish mutant, *ygl8*, was identified by screening of an EMS-induced mutant pool. By a map-based cloning method, the mutation of *ygl8* was shown to be located in a 54-kb genomic region between InDel marker ID-3 and SSR marker RM12339 on chromosome 1. Furthermore, a nucleotide mutation from C to T in *Loc_Os01g73450* was identified. By transgenic complementation and RNAi methods, *Loc_Os01g73450* was confirmed to be Ygl8. The nuclear-encoded chloroplast-targeted YGL8 protein contains a conserved UMP kinase domain and shares sequence similarity with UMP kinases of eukaryotic and prokaryotic (especially cyanobacterial) origins. The mutation of YGL8 had a negative impact on the expression of chloroplast genome-encoded thylakoid membrane constitutive genes, which affected the grana lamella structure and reduced the chlorophyll level, resulting in the yellowish phenotype, a reduced photosynthetic rate and poor yield characteristics. For further identification of the function and mechanism of action of the YGL8 protein, the following additional work is required: confirming the enzyme activity of YGL8; detecting and comparing the levels of UTP, UDP and UMP between the WT and the *ygl8* mutant; and testing the expression of other genes that may be regulated by YGL8.

## Methods

### Plant materials

The stably inherited mutation *ygl8* was derived from the progeny of an excellent *indica* restorer line Jinhui10 with seeds treated by 1 % EMS. Jinhui10 plants represent the Wild-type. Xinong1A, a cytoplasmic male sterile line bred by the Rice Research Institute of Southwest University, Chongqing, China, was crossed with *ygl8* and the F_1_ plants were self-crossed to generate the F_2_ mapping population to genetic analysis and gene mapping of *YGL8*. Rice plants were cultivated under natural conditions in an experimental field at the Southwest University Rice Research Institute.

### Physiological analysis

The first leaves from the top at the seedling stage, the tilling stage and heading stage of the WT and *ygl8* were collected and used to measure the levels of photosynthetic pigments [33]. During the heading stage, at 9:00 am on a sunny day, the first leaves from the top of the WT and *ygl8* were used to measure stomatal conductance, intercellular CO_2_ concentration, photosynthetic rate and transpiration rate with an LI-6400 portable photosynthesis meter (LiCor, Lincoln, NE, USA) in accordance with the manufacturer’s instructions. Values are the means ± SD of three biological repeats. The Student’s *t* test was applied for statistical analysis.

### Transmission electron microscopy

The leaf cellular structure of WT and *ygl8* plants was observed by TEM, as described by Wu et al. [6] with some modification. Leaves of WT and *ygl8* was collected from 3-week-old plants grown in the experiment filed under natural conditions and fixed in primary fixative solution (3.5 % glutaraldehyde) and then post-fixed for 2 h with 1 % osmium tetroxide after washing with 0.1 mol L^−1^ PBS buffer. Tissues were stained with uranyl acetate, dehydrated in ethanol and embedded in Spur’s medium prior to thin sectioning. Samples were stained again and examined using an H-7500 TEM (Hitachi, Japan).

### Survey of agronomic traits

During the mature stage, 5 WT and *ygl8* mutant plants, respectively, were used to measure plant height, effective panicle number per plant, grain number per panicle, filled-grain number per panicle, seed-setting rate and 1000-grain weight. Values are the means ± SD of three biological repeats. The Student’s *t* test was applied for statistical analysis.

### DNA extraction, primer design and PCR protocol

Bulked segregation analysis was employed for linkage analysis [34]. Total DNA of the parental lines or bulk DNA was extracted using the cetyl trimethyl ammonium bromide method [35], whereas genomic DNA of F_2_ individuals was isolated using an alkaline extraction method [36]. SSR markers were derived from http://archive.gramene.org/markers/microsat/ or identified by SSR Hunter. InDel primers were detected with Vector NTI software after genome re-sequenced between Xinong1A and Jinhui10. Newly developed markers used to map-cloning the *YGL8* gene were listed in Table S1 in the Additional file 1. The total PCR reaction volume was 12.5 μL and contained the following components: 1.25 μL of 10× PCR buffer, 1 μL 1 μmol L^−1^ primers, 1 μL of 50 ng μL^−1^ DNA, 0.75 μL of 25 mmol L^−1^ MgCl_2_, 0.5 μL of 2.5 mmol L^−1^ dNTPs, 7.9 μL of ddH_2_O, and 0.1 μL of 5 U μL^−1^*Taq* DNA polymerase. Amplifications were performed by the following procedure: 5 min at 94 °C, followed by 35 cycles of 30 s at 94 °C, 30 s at 55 °C and 1 min at 72 °C, with a final extension at 72 °C for 10 min. The PCR products were separated on 10 % polyacrylamide gels and visualised by rapid silver staining [37].

### Genetic analysis and linkage map construction

Recessive F_2_ individuals showed banding patterns similar to those of Xinong1A (designated A), the *ygl8* mutant (designated B) and the F_1_ hybrid between Xinong1A and *ygl8* (designated H). Total 11212 F_2_ plants (8400 WT and 2812 recessive mutant plants) were used to genetic analysis and linkage map construction. The phenotypic ratios of segregating F_2_ populations were evaluated using Pearson’s Chi-square test for goodness-of-fit to the 3:1 (wild-type: mutant) ratio expected for single gene recessive mutation. Percentage genetic distances were calculated using the formula [(*H* + 2*A*)/2*n*] × 100 and a linkage map was constructed accordingly. In this formula, *H* is the number of H-type plants, *A* is the number of A-type plants, and *n* is the total number of F_2_ recessive individuals used for gene mapping of *YGL8*.

### Vector construction and rice transformation

For complementation of the *ygl8* mutation, a 6478-bp genomic fragment that contains 3844-bp Ygl8 genomic sequence including introns and extrons, coupled with 1570-bp upstream and 1054-bp downstream sequences, was amplified with YGL8com-F and YGL8com-R. The resulting PCR products was digested with *BamHI* and *PstI*, and cloned in to the binary vector Pcambia1301. The resulting recombinant plasmids were transformed into *ygl8* by *A. tumefaciens*-mediated transformation method as described previously [36]. To construct a RNAi vector for *YGL8*, a 334-bp fragment of *YGL8* complementary DNA was amplified with the primers YGL8RNAi-F1 and YGL8RNAi-R1. The resulting PCR product was digested with *BamHI* and *KpnI* and inserted into vector pTCK303 [38] to obtain the intermediate vector. The PCR products amplified by YGL8RNAi-F2 and YGL8RNAi-R2 was digested with *SacI* and *SpeI* and cloned into the intermediate vector. The recombinant plasmids were transformed into Zhonghua11 plants by the *A. tumefaciens*-mediated transformation method. The primer sequences were listed in Table S2 (Additional file 3).

### Multiple sequence alignment and phylogenetic tree construction

Protein sequences applied in multiple sequence alignment and phylogenetic tree construction were acquired by searching Phytozome (http://phytozome.jgi.doe.gov/pz/portal.html) using the YGL8 sequence as a query. Multiple sequence alignment was performed with ClustalX software using multiple alignment modes. Phylogenetic tree was constructed by MEGA 5.2 with the maximum likelihood method with JTT model. The bootstrap support values for each node were from 500 replications and shown next to the branches [19].

### Subcellular location

The full-length coding sequence of YGL8 was amplified with YGL8SL-F and YGL8SL-R, the resulting PCR products were digested with the *Xho*I and *Spe*I and cloned into vector pA7-GFP. Both pA7-YGL8-GFP and empty pA7-GFP vector were transformed into rice protoplasts [19]. GFP fluorescence was detected using a confocal laser scanning microscope (Olympus FluoView FV1000; Japan). YGL8SL-F and YGL8SL-R were listed in Table S2 (Additional file 3).

### Quantitative real-time PCR

Total rice RNA was extracted from the roots, culms, leaf sheaths, flag leaves, young panicles (white) and green panicles at the heading stage using an RNAprep Pure Plant kits (Tiangen Co. Ltd., China). First-strand cDNAs were synthesized using a PrimeScript RT Reagent Kit with gDNA Eraser (Perfect Real Time) (Takara Bio Inc., China). qRT-PCR was performed using the SYBR Premix Ex Taq II (Tli RNaseH Plus) kit (Takara Bio Inc., China) in an ABI Prism 7500 Real-Time PCR System (Invitrogen, USA). Relative expression levels of genes were calculated using the formula 2^-ΔΔCt^ and are expressed relative to *OsActin1*. Values are the means ± SD of three biological repeats. The Student’s *t* test was applied for statistical analysis. The qRT-PCR primers used in this study was listed in Table S3 in the Additional file 3.

## Abbreviations

CAO, chlorophyllide a oxygenase; CDS, coding sequence; Chl *a*, chlorophyll *a*; Chl *b*, chlorophyll *b*; Chl, chlorophyll; CHLD, Mg chelatase D subunit; CHLG, chlorophyll synthase; CHLM, Mg protoporphyrin methyltransferase; DVR, 3,8-Divinyl reductase protochlorophyll α-8-vinyl reductase; EMS, ethyl methane sulfonate; HEMA, glutamyl-tRNA reductase; HEMC, hydroxymethylbilane synthase; HEMD, uroporphyrinogen III synthase; HEME, uroporphyrinogen decarboxylase; InDel markers, insertion/deletion markers; JTT model, Jones-Taylor-Thornton model; NADPH, pchlide oxidoreductase; ORFs, open reading frames; PORA, NADPH:protochlorophyllide oxidoreductase A; PsaA, photosystem I P700 chlorophyll a apoprotein A1; PsaB, photosystem I P700 chlorophyll a apoprotein A2; QRT-PCR, quantitative real time-polymerase chain reaction; rbcL, rubisco large subunit; RNAi, RNA interference; SSR, simple sequence repeat; TEM, transmission electron microscopy; UDP, uridine diphosphate; UMP, uridine monophosphate; UMP kinase, uridine monophosphate kinase; UTP, uridine triphosphate.

## Additional files

Additional file 1: Statistics of agronomic traits of the wild-type and *ygl8* mutant. (PPTX 36 kb) Additional file 2: Statistics of photosynthetic parameters of *ygl8* mutant in comparison to the wild-type. (PPTX 36 kb) Additional file 3: Primer sequences used in this study. (DOC 61 kb)
