# Supplementary material for: Map-based cloning and functional analysis of YGL8, which controls leaf colour in rice (Oryza sativa)
Source: BMC Plant Biol. 2016 Jun 13;16:134. doi: 10.1186/s12870-016-0821-5 (PMC4907030; doi:10.1186/s12870-016-0821-5)
Supplement: Additional file 1: — Statistics of agronomic traits of the wild-type and ygl8 mutant. (PPTX 36 kb) [file 12870_2016_821_MOESM1_ESM.pptx]

## Slide 1
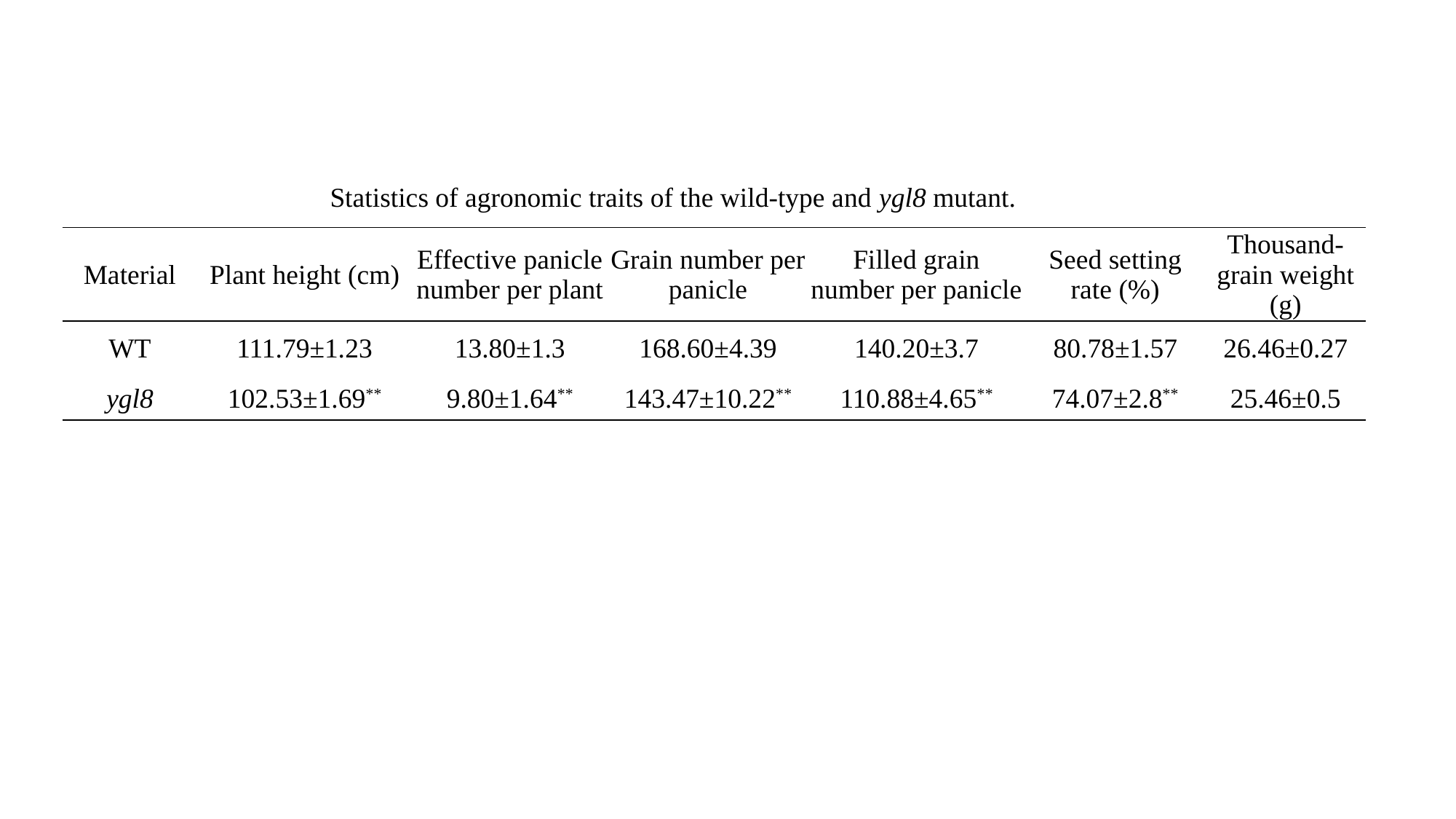

Statistics of agronomic traits of the wild-type and ygl8 mutant.
| Material | Plant height (cm) | Effective panicle number per plant | Grain number per panicle | Filled grain number per panicle | Seed setting rate (%) | Thousand-grain weight (g) |
| --- | --- | --- | --- | --- | --- | --- |
| WT | 111.79±1.23 | 13.80±1.3 | 168.60±4.39 | 140.20±3.7 | 80.78±1.57 | 26.46±0.27 |
| ygl8 | 102.53±1.69\*\* | 9.80±1.64\*\* | 143.47±10.22\*\* | 110.88±4.65\*\* | 74.07±2.8\*\* | 25.46±0.5 |
