# Supplementary material for: Map-based cloning and functional analysis of YGL8, which controls leaf colour in rice (Oryza sativa)
Source: BMC Plant Biol. 2016 Jun 13;16:134. doi: 10.1186/s12870-016-0821-5 (PMC4907030; doi:10.1186/s12870-016-0821-5)

## Slide 1
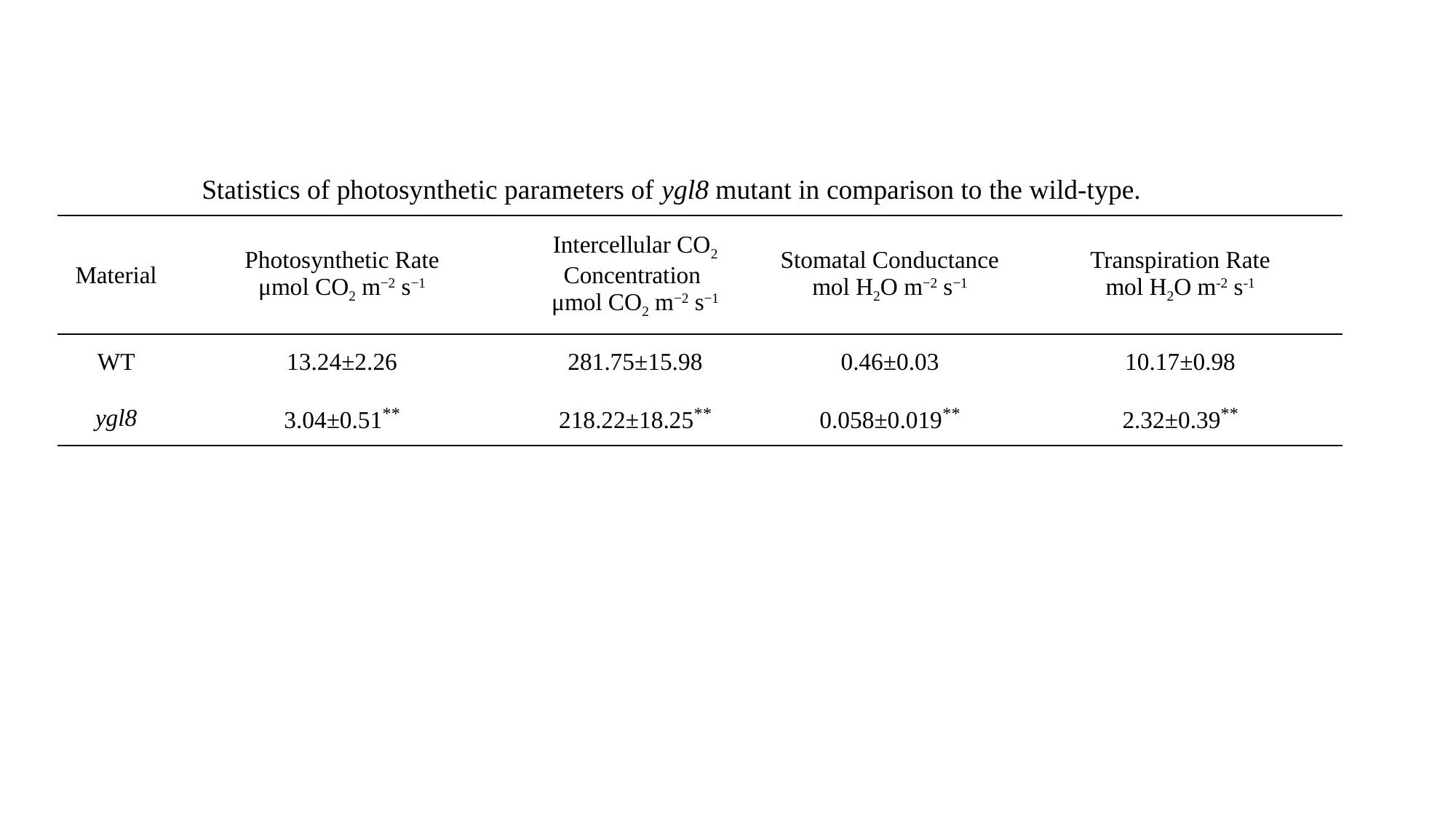

Statistics of photosynthetic parameters of ygl8 mutant in comparison to the wild-type.
| Material | Photosynthetic Rate μmol CO2 m−2 s−1 | Intercellular CO2 Concentration μmol CO2 m−2 s−1 | Stomatal Conductance mol H2O m−2 s−1 | Transpiration Rate mol H2O m-2 s-1 |
| --- | --- | --- | --- | --- |
| WT | 13.24±2.26 | 281.75±15.98 | 0.46±0.03 | 10.17±0.98 |
| ygl8 | 3.04±0.51\*\* | 218.22±18.25\*\* | 0.058±0.019\*\* | 2.32±0.39\*\* |

## Slide 2
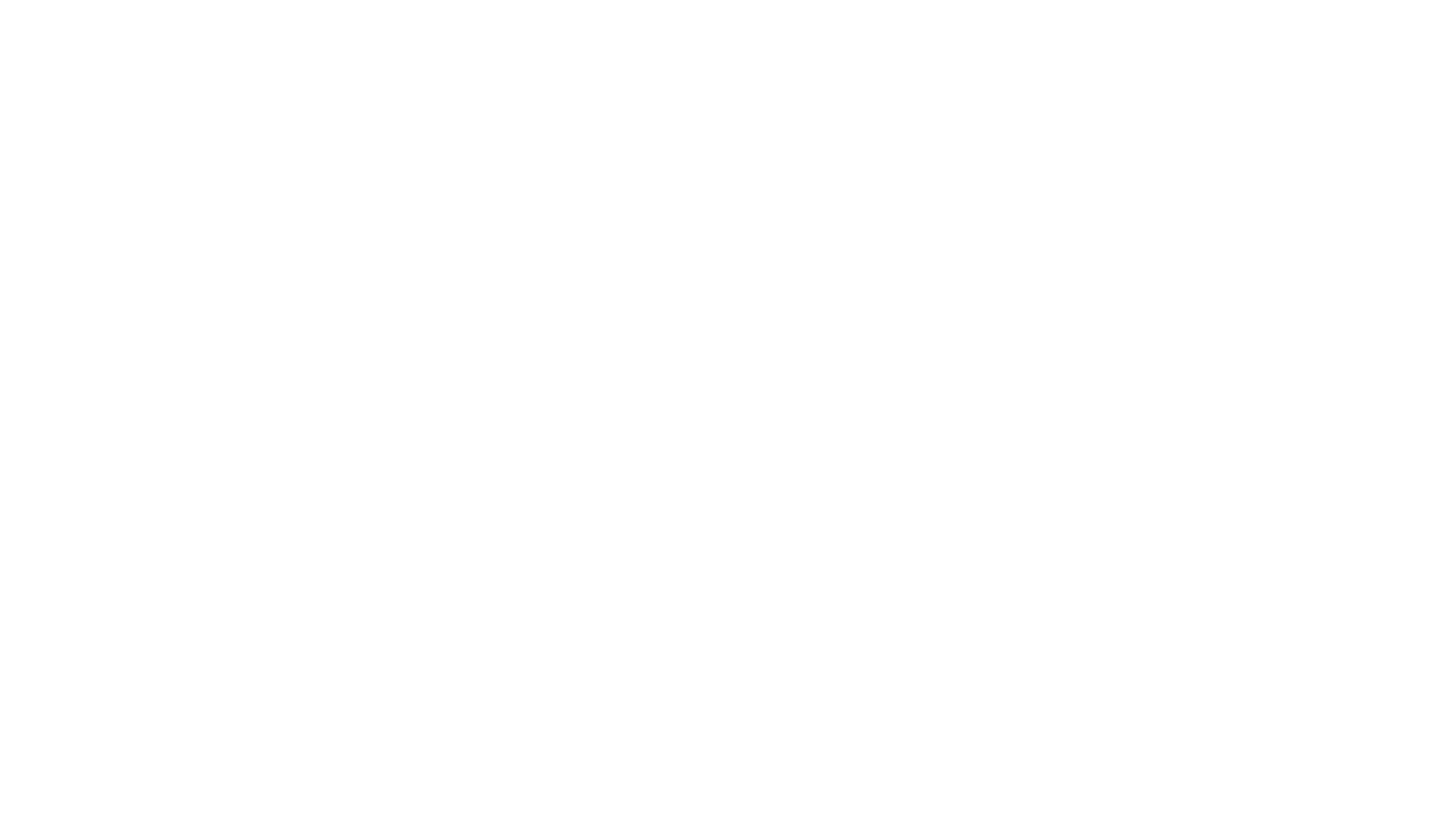

Supplement: Additional file 2: — Statistics of photosynthetic parameters of ygl8 mutant in comparison to the wild-type. (PPTX 36 kb) [file 12870_2016_821_MOESM2_ESM.pptx]
