# Supplementary material for: Map-based cloning and functional analysis of YGL8, which controls leaf colour in rice (Oryza sativa)
Source: BMC Plant Biol. 2016 Jun 13;16:134. doi: 10.1186/s12870-016-0821-5 (PMC4907030; doi:10.1186/s12870-016-0821-5)
Supplement: Additional file 3: — Primer sequences used in this study. (DOC 61 kb) [file 12870_2016_821_MOESM3_ESM.doc]

Table S1. Primers used to map-based clone Ygl8 gene

| Marker | Forward primer | Revrse primer |
| --- | --- | --- |
| RM6489 | GGACCTCCCGGTATTGGATAC | CCAGAAACCCTCACACAGCG |
| ID3 | TGGAACGGAGGGAGTAGTTAGCT | TTCCTCGCCGATGCTCTCTC |
| ID8 | CTGATGGTGGTGGGCAATGT | CCACCTATCATCCCCTCCATC |
| ID13 | AGAGATTCCAAGGCCTCGTTG | GGTACACAATACAGTGAGCCCC |
| RM12339 | GCGAGCCATCACCAACTACG | CACCATGAGCTGCTGGATCA |

Table S2. Primers used to construct complentation vector, RNAi and subcellular location vector

| Primer | Sequences | Restriction enzyme |
| --- | --- | --- |
| YGL8COM-F | GCC ***GGATCC*** GCCAAGGCTGGGTGCGGAATAAGTAC | *BamHI* |
| YGL8COM-R | GCC ***CTGCAG*** GGTTAGTGTAATAGCCTCCCGAGGTG | *PstI* |
| YGL8RNAi-F1 | GCC ***GGATCC*** CTCCAACTTTGGAGGTCAAAC | *BamHI* |
| YGL8RNAi-R1 | GCC ***GGTACC*** CATCACAGTAGCCAACATGCC | *KpnI* |
| YGL8RNAi-F2 | GCC ***GAGCTC*** CTCCAACTTTGGAGGTCAAAC | *SacI* |
| YGL8RNAi-R2 | GCC ***ACTAGT*** CATCACAGTAGCCAACATGCC | *SpeI* |
| YGL8SL-F | GCC ***CTCGAG*** ATGGCCGCCGCCGCCGCCGCCGCC | *XhoI* |
| YGL8SL-R | GCC ***ACTAGT*** TAACTCGTTCACCAATCTTC | *SpeI* |

Table S3. QRT-PCR primers used in the present study

| Gene | Forward primer | Reverse primer | Reference |
| --- | --- | --- | --- |
| YGL8 | GATTACATTGGCATGTTGGCTAC | CTGCATTGATTTCTGCACAACG |  |
| HEMA | CGCTATTTCTGATGCTATGGGT | TCTTGGGTGATGATTGTTTGG | [1] |
| HEMC | TGCTTGACTGCAAGTTCCCTTG | CTAGAGCCAACAATGTAGCATGG |  |
| HEMD | TGGAAGGCTGCTGGAAATCCTAAG | TCCTTGGAAGCTCTGAGGCCAA |  |
| HEME | AGGAGCAGGTGAGGGAGCT | TGGCGTCTGCAAGGTGAGAC |  |
| CHLD | GCTTGCAGAAAGCTACACAAGC | AGGCCGTGAGCTAAAGGAGA | [2] |
| CHLM | CCATCCATTGGTCTCCTTATGACA | GTAGCCTACTTACCATCAATGAGTC | [2] |
| DVR | CAGGTCGAGACCGTCAAGAAC | ATGACCTGGATCGGCACCTTG | [2] |
| PORA | TGTACTGGAGCTGGAACAACAA | GAGCACAGCAAAATCCTAGACG | [3] |
| CHLG | ATGAACCTTACCGTCCTATTCCTT | GGACCCACCAACAGCAAGAT |  |
| CAO | GATCCATACCCGATCGACAT | CGAGAGACATCCGGTAGAGC | [1] |
| PsaA | GCGAGCAAATAAAACACCTTTC | GTACCAGCTTAACGTGGGGAG | [1] |
| PsaB | CGAACCTATATTTGCTCAATGGATAC | GATGAACCAAGAAATCTCCAGGTC |  |
| PsbA | GCGGTTCCCTATTCAGTGCTATG | TAACCATGAGCGGCCACAATATT |  |
| PsbB | TAGTTTCTGGTTGGGCTGGCTC | CTCCAACCACCCCACGAATTG |  |
| PsbC | GTTCCCCAACGGGAGAGGTTAT | GAGCCTAAAGGAGCATGGGTCAT |  |
| PsbE | TGTCTGGAAGCACGGGAGAACGT | GTTTGGCCGAGGACTTCCAAACAC |  |
| PsbF | CTATTTTTACAGTGCGATGGCTGG | TATCGTTGGATGAACTGCATTGCT |  |
| petA | GCAGCAAGGTTATGAAAACCCAC | AACAGCACCCACATTCAACCCT |  |
| PetB | TTCAGACCTCGCAACCAGACTG | AACAAAAGGCAAGGGTTCTTCGA |  |
| PetD | GCGTGGCCCAACGATCTTTTAT | TCCAGAGGAGTTGCAAACGGAT |  |
| PetG | TCCCCTCGAACAAATCGAAATG | CAACTGATCCCCACGTCTGTATT |  |
| AtpA | TGAATCTCCTGCTCCGGGTATAAT | TGCTGTTTTGCCGGTTTGTCT |  |
| AtpB | TCGCAATTCTTGGGTTGGATGA | CAACATACTTTCCCGGAGAACCG |  |
| rbcL | CTTGGCAGCATTCCGAGTAA | ACAACGGGCTCGATGTGATA | [3] |
| Actin | GACCCAGATCATGTTTGAGACCT | CAGTGTGGCTGACACCATCAC | [4] |

**References**

[1] Kang ZH, Li GR, Huang JL, Niu XD, Zou HY, Zang GC, Wenwen Y, Wang GX. Photosynthetic and physiological analysis of the rice high-chlorophyll mutant (*Gc*). Plant Physiol and Biochem. 2012;60:81-87.

[2] Shi J, Wang Y, Guo S, Ma L, Wang Z, Zhu X, Sang X, Ling Y, Wang N, Zhao F, He G. Molecular Mapping and Candidate Gene Analysis of a *Yellow-Green Leaf 6* (*ygl6*) Mutant in Rice. Crop Science. 2015;55(2):669-680.

[3] Wu ZM, Zhang X, He B, Diao LP, Sheng SL, Wang JL, Guo XP, Su N, Wang LF, Jiang L, Wang CM, Zhai HQ, Wan JM. A Chlorophyll-Deficient Rice Mutant with Impaired Chlorophyllide Esterification in Chlorophyll Biosynthesis. Plant Physiol. 2007;145(1):29-40.

[4] Ren DY, Li YF., Zhao FM, Sang XC, Shi JQ, Wang N, Guo S, Ling YH, Zhang CW, Yang ZL, He GH. *MULTI-FLORET SPIKELET1*, which encodes an AP2/ERF protein, determines spikelet meristem fate and sterile lemma identity in rice. Plant Physiol. 2013;162(2):872-884.
